# Impact of foliar application of some metal nanoparticles on antioxidant system in oakleaf lettuce seedlings

**DOI:** 10.1186/s12870-020-02490-5

**Published:** 2020-06-23

**Authors:** Rita Jurkow, Robert Pokluda, Agnieszka Sękara, Andrzej Kalisz

**Affiliations:** 1 Department of Horticulture, University of Agriculture in Krakow, 29 Listopada 54, 31-425 Kraków, Poland; 2 Department of Vegetable Sciences and Floriculture, Mendel University in Brno, Valtická 337, 691 44 Lednice, Brno, Czech Republic

**Keywords:** Antioxidants, Chlorophyll, Gold, *Lactuca sativa* L. var. *foliosa*, Nanometals, Platinum, Silver

## Abstract

**Background:**

Nanoparticles (NPs) serve various industrial and household purposes, and their increasing use creates an environmental hazard because of their uncontrolled release into ecosystems. An important aspect of the risk assessment of NPs is to understand their interactions with plants. The aim of this study was to examine the effect of Au (10 and 20 ppm), Ag, and Pt (20 and 40 ppm) NPs on oakleaf lettuce, with particular emphasis on plant antioxidative mechanisms. Nanoparticles were applied once on the leaves of 2-week-old lettuce seedlings, after next week laboratory analyses were performed.

**Results:**

The antioxidant potential of oakleaf lettuce seedlings sprayed with metal NPs at different concentrations was investigated. Chlorophylls, fresh and dry weight were also determined. Foliar exposure of the seedlings to metal NPs did not affect ascorbate peroxidase activity, total peroxidase activity increased after Au-NPs treatment, but decreased after applying Ag-NPs and Pt-NPs. Both concentrations of Au-NPs and Pt-NPs tested caused an increase in glutathione (GSH) content, while no NPs affected L-ascorbic acid content in the plants. Ag-NPs and Pt-NPs applied as 40 ppm solution increased total phenolics content by 17 and 15%, respectively, compared to the control. Carotenoids content increased when Ag-NPs and Au-NPs (20 and 40 ppm) and Pt-NPs (20 ppm) were applied. Plants treated with 40 ppm of Ag-NPs and Pt-NPs showed significantly higher total antioxidant capacity and higher concentration of chlorophyll *a* (only for Ag-NPs) than control. Pt-NPs applied as 40 ppm increased fresh weight and total dry weight of lettuce shoot.

**Conclusions:**

Results showed that the concentrations of NPs applied and various types of metal NPs had varying impact on the antioxidant status of oakleaf lettuce. Alteration of POX activity and in biosynthesis of glutathione, total phenolics, and carotenoids due to metal NPs showed that tested nanoparticles can act as stress stimuli. However, judging by the slight changes in chlorophyll concentrations and in the fresh and dry weight of the plants, and even based on the some increases in these traits after M-NPs treatment, the stress intensity was relatively low, and the plants were able to cope with its negative effects.

## Background

Nanoparticles (NPs) are materials with at least two dimensions between 1 nm and 100 nm [1]. The unique properties of nanoparticles result from their extremely small size and large surface to volume ratio, which lead to differences in their mechanical and biological properties, catalytic activity, thermal and electrical conductivity, optical absorption, and melting point in comparison to larger particles of the identical chemical composition [1]. The effects of nanoparticles on plants have been the focus of many studies, which have showed their phytotoxicity or beneficial effects or demonstrated no consequential responses in the plants [2]. Still, little is known about the impacts of specific nanoparticles at given concentrations on specific plant species [3].

It is reported that nearly 25% of all nanotechnology consumer products contain silver nanoparticles (Ag-NPs) because of their antibacterial and antifungal properties; in agriculture Ag-NPs are mainly used for plant disease management [4]. Gold nanoparticles (Au-NPs) are common in household, industrial, and healthcare products [5]. Platinum-based nanomaterials have been shown to be excellent therapeutic agents and they are frequently used in chemotherapy [6]. At nanoscale platinum nanoparticles (Pt-NPs) are suitable for designing new electrochemical sensors and biosensors [7]. However, a side effect of using nanotechnology is the possible release of nanomaterials into the environment, thus understanding of their interactions within ecosystems, including plants, is necessary.

The impact of a chemical element on plants in the form of nanoparticles can be stronger compared to that of its corresponding bulk counterpart and this impact can be both positive and negative [2]. Stampoulis [8] observed that Ag content in zucchini shoots was an average 4.7 times higher in plants exposed to 10–1000 mg L^− 1^ Ag-NPs than those treated with bulk Ag powder at similar concentrations, due to higher levels of ion release from Ag-NPs. Easy penetration of NPs into plants and specific features of nanoparticles can cause strong reaction at various levels, including alterations in metabolic processes. Kumari et al. [9] treated *Allium cepa* cells with Ag-NPs and noticed different kinds of chromosomal aberrations, such as stickiness, chromosomal breaks, gaps, disturbed metaphase, and cell wall disintegration. The phytotoxicity effects of Ag-NPs on plants at the morphological and physiological level were described in detail by Yan and Chen [10]. Au-NPs accumulate inside the plant tissues due to exposure to metal nanoparticles, but Au-NPs uptake is believed to be size selective [11]. Plants exposed to Au-NPs exhibited both positive and negative effects, which were summarized by Siddiqi and Husen [12]. Arora et al. [13] observed that Au-nanoparticle treatment positively affected various growth- and yield-related parameters of *Brassica juncea*. On the other hand, Feichtmeier et al. [14] noted that fresh biomass of barley decreased with increasing concentration of Au-NPs. Asztemborska et al. [15] found that *Lepidium sativum* and *Sinapis alba* were able to take up Pt-NPs from the growth medium and translocate them to shoots. There is not much information about the phytotoxicity of Pt-NPs on plants, but Shiny et al. [16] did not observe harmful effects of Pt-NPs on tomato and radish seeds germination.

Nanoparticles can interfere with electron transport chains in mitochondria and chloroplasts, which may result in an oxidative burst, followed by the release of reactive oxygen species (ROS) in cell compartments [1, 5]. For example, Jiang et al. [17] established size-dependent ROS generation caused by titanium nanoparticles (TiO_2_-NPs). Plants developed antioxidant mechanisms to control the level of ROS and maintain ROS scavenging processes in balance [18]. These mechanisms involve antioxidants enzymes and non-enzymatic compounds which help plants to cope with stress [19]. Exposure to Ag-NPs can lead to oxidative stress in plants [10]. For example, Thiruvengadam et al. [20] found that Ag-NPs in higher concentrations caused excessive generation of superoxide radicals, increased H_2_O_2_ production, and lipid peroxidation in turnip seedlings. It has been suggested that the evidence of Au-NPs-mediated ROS generation in *B. juncea* seedlings is due to the increase in H_2_O_2_ content together with higher overall antioxidant activity [21]. While it has been shown that plants treated with acute high NPs doses exhibit oxidative stress and overproduction of ROS, which is evidence of NPs cytotoxicity, there is little research examining the effects of exposure to NPs at low doses, which could be safer and more environmentally relevant [22]. High-dose NPs exposure usually results in ROS overproduction and therefore cytotoxicity; low-dose exposure may lead to non-toxic modulation of redox signalling, which may cause an increase in plants’ stress tolerance. For this reason, we undertook to investigate the effects of Ag, Au and Pt nanoparticles, applied at different concentrations to the leaves, on the antioxidant status of oakleaf lettuce plants, as well as on possible alterations in the amount of fresh and dry weight and the chlorophyll content. We decided to analyse the impact of different concentrations of a given nanoparticle on the plant, but also compare specific effects of Ag, Au and Pt NPs on plant metabolism.

## Results and discussion

There was an increase in shoot fresh weight (FW) and total dry weight of oakleaf lettuce as a results of foliar spraying with 40 ppm Pt-NPs (Table 1). Other nanometals and concentrations used did not change these plant traits. The results published by Salama [23] for Ag-NPs at 60 ppm showed an increase by 30% in FW and by 27% in dry weight for common bean seedlings over control plants; in the case of corn seedlings at the same Ag-NPs concentration the corresponding values were 35 and 33% higher. Only at the highest concentration of 100 ppm, did the fresh and dry weights of both plant species decrease. As was observed by El-Batal et al. [24], for common bean seedlings the foliar application of Ag-NPs (5–60 ppm) significantly increased total fresh and dry weight per plant. However, there are other reports in which the action of Ag nanoparticles are negative in this respect. Mirzajani et al. [25] concluded that treatment of rice plants with different concentrations of Ag-NPs (0.30–60 mg L^− 1^) linearly and significantly decreased dry weight accumulation. Vannini et al. [26] observed a decrease in FW of germinating wheat seedlings treated with Ag-NPs (10 mg L^− 1^) and they suggested that such an effect was due to the release of Ag ions from Ag-NPs. Ag-NPs phytotoxicity has been demonstrated in several studies, but usually after root exposure to high concentrations of NPs. The Larue et al. [27] study is interesting because foliar exposure of lettuce seedlings to Ag-NPs did not lead to detectable phytotoxicity symptoms even at very high concentrations (from 10 mg L^− 1^ up to 1000 mg L^− 1^); we also did not observe any negative effects of applying Ag-NPs to oakleaf lettuce. Kumar et al. [28] found that total FW of *Arabidopsis thaliana* seedlings was increased by 3.7 and 6.3 times on their exposure to 10 and 80 μg mL^− 1^, respectively, of Au-NPs, in comparison to control. Conversely, the opposite results were obtained by Feichtmeier et al. [14] for barley seedlings, where fresh biomass per plant decreased with exposure to increasing concentrations of Au-NPs (3 to 10 μg mL^− 1^), but a concentration of 1 μg mL^− 1^ of Au-NPs in the nutrient medium had a stimulating effect on biomass. Astafurova et al. [29] observed a significant increase in the weight of wheat seedlings treated with Pt-NPs in both water and soil culture; however, dry weight of shoots increased only when the plants grew in one of two soil types tested. An increase in fresh weight and dry weight of lettuce shoot was observed in our experiment due to Pt-NPs applied at higher concentration. In our opinion, Pt can play a catalytic role in plant growth regulation processes when it is present at cells in a concentration sufficient to perform such a role [30].

**Table 1:** Leaf fresh weight and total dry weight of oakleaf lettuce seedlings depending on engineered nanoparticles (nano-metals; M-NPs) applied on the leaves in different concentrations as aqueous colloidal solutions. Control plants were sprayed with deionized water

M-NPs and concentration		Fresh weight (g per shoot)	Total dry weight (g per shoot)
Ag	20 ppm	4.071 ± 0.507	0.146 ± 0.018
Ag	40 ppm	3.911 ± 0.551	0.143 ± 0.022
Au	10 ppm	4.175 ± 0.470	0.138 ± 0.049
Au	20 ppm	4.229 ± 0.578	0.148 ± 0.015
Pt	20 ppm	4.047 ± 0.359	0.138 ± 0.018
Pt	40 ppm	4.248 ± 0.267 *	0.161 ± 0.015 *
Control		3.472 ± 0.407	0.130 ± 0.008

*Denotes significant differences (*p* ≤ 0.05) between particular nanometal and unexposed control, means for concentration of given nanometal with no letters are not significantly different at *p* ≤ 0.05, comparisons were performed by Fisher’s LSD test. Each value represents the mean ± SD

**Fig. 1:**
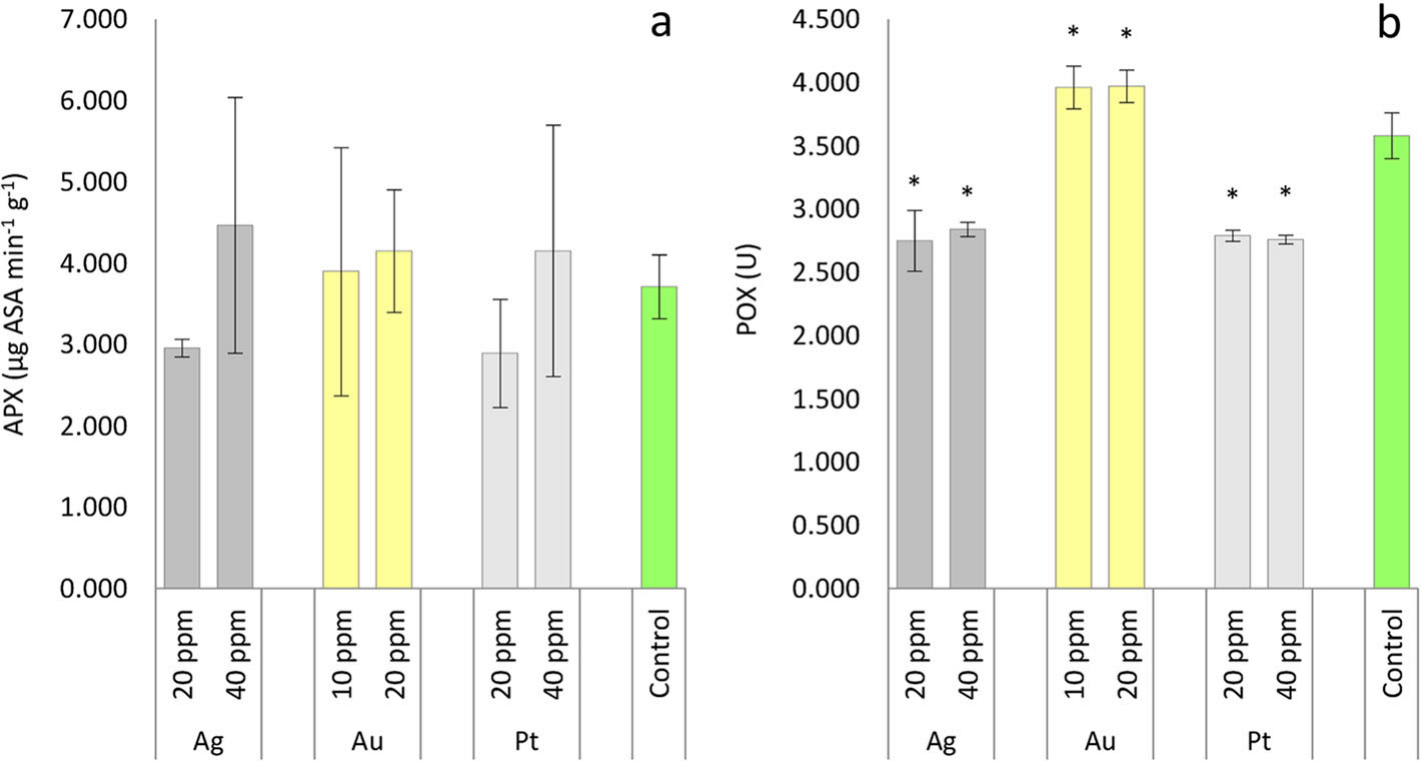
Ascorbate peroxidase (**a**) and total peroxidase (**b**) activity in oakleaf lettuce seedlings affected by Ag, Au, and Pt nanoparticles applied to the leaves in different concentrations as aqueous colloidal solutions. Control plants were sprayed with deionized water. *Denotes significant differences (*p* ≤ 0.05) between particular nanometal and unexposed control, means for concentration of given nanometal with no letters are not significantly different at *p* ≤ 0.05, comparisons were performed by Fisher’s LSD test. Bars represent standard deviations (± SD)

Although the results presented showed some variation in APX activity in oakleaf lettuce seedlings treated with nanoparticles, this was not confirmed statistically, most of the variable values were strongly dispersed around the average (Fig. 1a). Plants treated with 10 and 20 ppm of Au-NPs showed significantly higher POX activity then control seedlings, while Ag-NPs and Pt-NPs decreased activity of POX in the plants (Fig. 1b). In general, nanoparticle treatment was reported to be responsible for the increment of enzymatic activities in treated plants, but the degree of this increase was dependent on the concentration of applied nanoparticles and on the type of NPs [31]. The results obtained by Lei et al. [32] clearly showed that nano-TiO_2_ treatment could significantly increase the activity of several enzymes, including superoxide dismutase, catalase, APX, and guaiacol peroxidase (GPX), in spinach plants. Data obtained by Homaee and Ehsanpour [33] for potato plantlets showed that APX activity increased due to Ag-NPs treatment. In other work, when 25–400 ppm Ag-NPs were tested on *B. juncea*, ascorbate peroxidase activity was highest at the highest concentrations of Ag-NPs showing only slight depression at 50 ppm in comparison to the control [34]. Kumar et al. [28] noted that APX activity was 1.24- and 1.78-fold higher in *A. thaliana* seedlings exposed to 10 and 80 μg mL^− 1^ of Au-NPs, respectively, than in control. Gunjan et al. [21] observed in *B. juncea* seedlings a marginal variation in APX activity with Au-NPs at 200 ppm concentration, but further increase in the concentration of nanoparticles contributed a remarkable increase in APX activity with a maximum at 400 ppm. Scientific reports are quite consistent in terms of increasing APX activity due to the application of nanoparticles, but this phenomenon was not observed in the current experiment. Krishnaraj et al. [35] reported significant increase in POX activity in the leaves of *Bacopa monnieri* plants subjected to 10 ppm Ag-NPs, which is not consistent with our results. According to Sharma et al. [34], the activity of GPX in *B. juncea* seedlings increased continuously with increasing concentrations of Ag-NPs from 25 ppm to 400 ppm. In mustard plants Gunjan et al. [21] showed a simultaneous increase in GPX activity with increasing concentrations of Au-NPs and GPX activity at 400 ppm was 1.28-fold greater than in the control plants. Such an effect of Au-NPs on POX activity is in agreement with our findings. Such increase in this enzyme activity may be due to the stress over the seedlings of oakleaf lettuce imposed by Au-NPs, which is connected with detoxifying overproduced reactive oxygen species (ROS). To the best of our knowledge, the effects of Pt-NPs on antioxidant enzyme activity are not described in scientific reports. The action of enzymatic mechanisms and non-enzymatic antioxidants in overcoming stress by plants is interrelated. Our study demonstrated that the content of non-enzymatic antioxidants often increased in plants after treatment with Ag-NPs and Pt-NPs, so mainly these type of compounds participated in the detoxification of ROS.

**Fig. 2:**
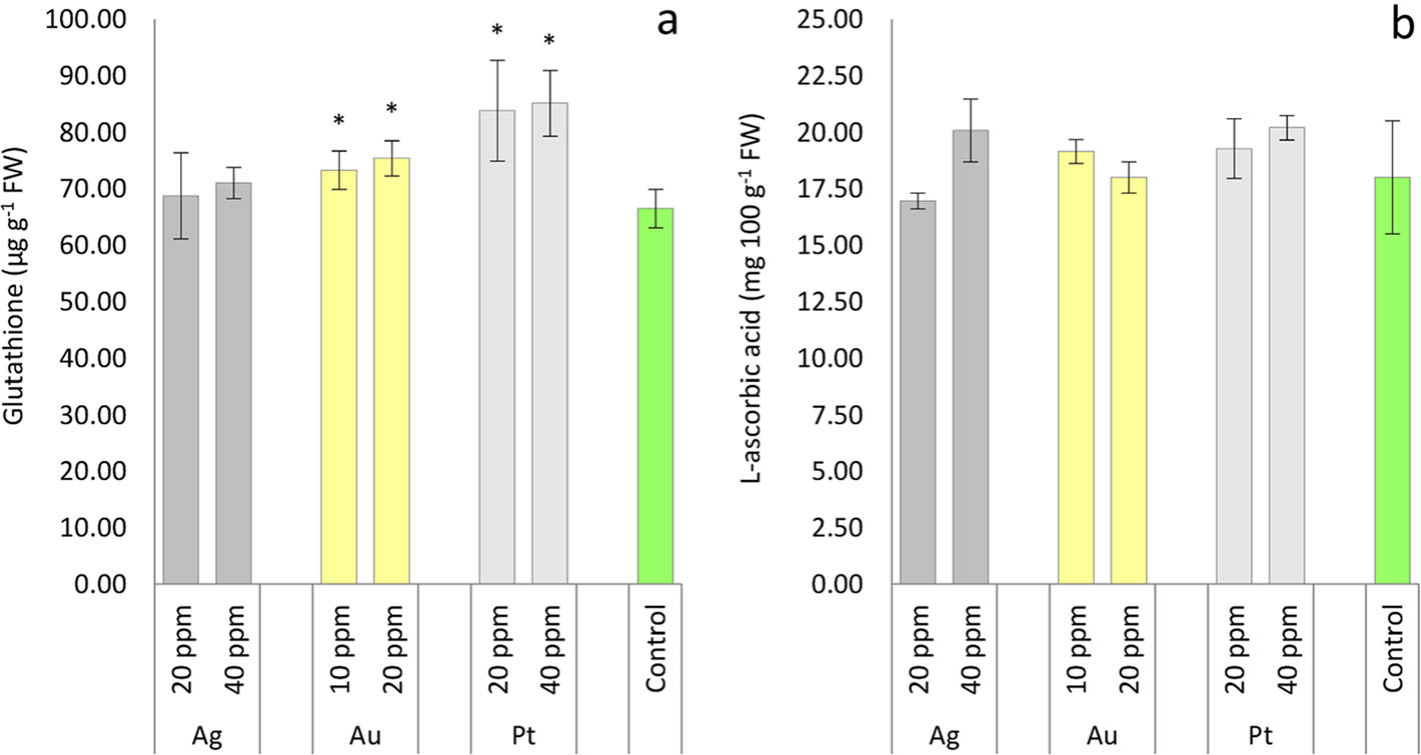
Content of glutathione (**a**) and L-ascorbic acid (**b**) in oakleaf lettuce seedlings affected by Ag, Au and Pt nanoparticles applied to the leaves in different concentrations as aqueous colloidal solutions. Control plants were sprayed with deionized water. *Denotes significant differences (*p* ≤ 0.05) between particular nanometal and unexposed control, means for concentration of given nanometal with no letters are not significantly different at *p* ≤ 0.05, comparisons were performed by Fisher’s LSD test. Bars represent standard deviations (± SD)

We observed that foliar application of Au-NPs and Pt-NPs significantly increased glutathione (GSH) content in oakleaf lettuce seedlings compared to control (Fig. 2a). The differences in glutathione level reached 26% for plants treated with 20 ppm Pt-NPs and 28% for 40 ppm Pt-NPs more than those of control plants, the corresponding values for Au-NPs were 10% (10 ppm) and 13% (20 ppm). The Ag nanoparticles did not stimulate glutathione biosynthesis in oakleaf lettuce. The Ag, Au, and Pt nanoparticles did not influence L-ascorbic acid concentration in the plants compared to the control (Fig. 2b). Homaee and Ehsanpour [33] noted that, compared to the control, no alteration was observed in GSH or ascorbate concentration at 2 mg L^− 1^ Ag-NPs treatment in potato plantlets. However, a significant reduction in the content of these compounds were seen in plantlets exposed to higher concentrations of Ag-NPs. According to these authors, Ag ions released from Ag-NPs have a high affinity to the sulfhydryl groups of biomolecules, GSH might be targeted by Ag ions and thus drained from the cells. This drainage was possible explanation for the lack of significant changes in GSH concentration in lettuce plants treated with Ag-NPs in our experiment. In the case of *A. thaliana*, the up-regulation of genes involved in glutathione synthesis was observed when plants were treated with 0.2–1 mg L^− 1^ Ag-NPs [36]. After Ag-NPs exposure a large increase in ascorbic acid content was observed in *Asparagus officinalis* [37]. The effects of M-NPs on glutathione and L-ascorbic acid concentration in the plants have been partially examined for Ag-NPs only, as can be seen in this paragraph. To the best of our knowledge, the effects of Au-NPs and Pt-NPs on these compounds have not yet been presented, and the mechanism of increase in glutathione content due to the action of these nanoparticles, observed in the present experiment, requires further in-depth research. Both, ascorbate (AsA) and GSH are connected to the reactions network, the ascorbate-glutathione (AsA-GSH) pathway. AsA-GSH show delicate balance and possible changes in glutathione and ascorbate are not directly proportional to each other, especially in conditions of oxidative stress resulted from metal/metalloid application [38]. In the present research, possibly alteration in AsA-GSH pathway caused by Au and Pt nanometals, but not Ag, led to an increase in the content of glutathione connected with lack of M-NPs effect in L-ascorbic acid concentration.

**Fig. 3:**
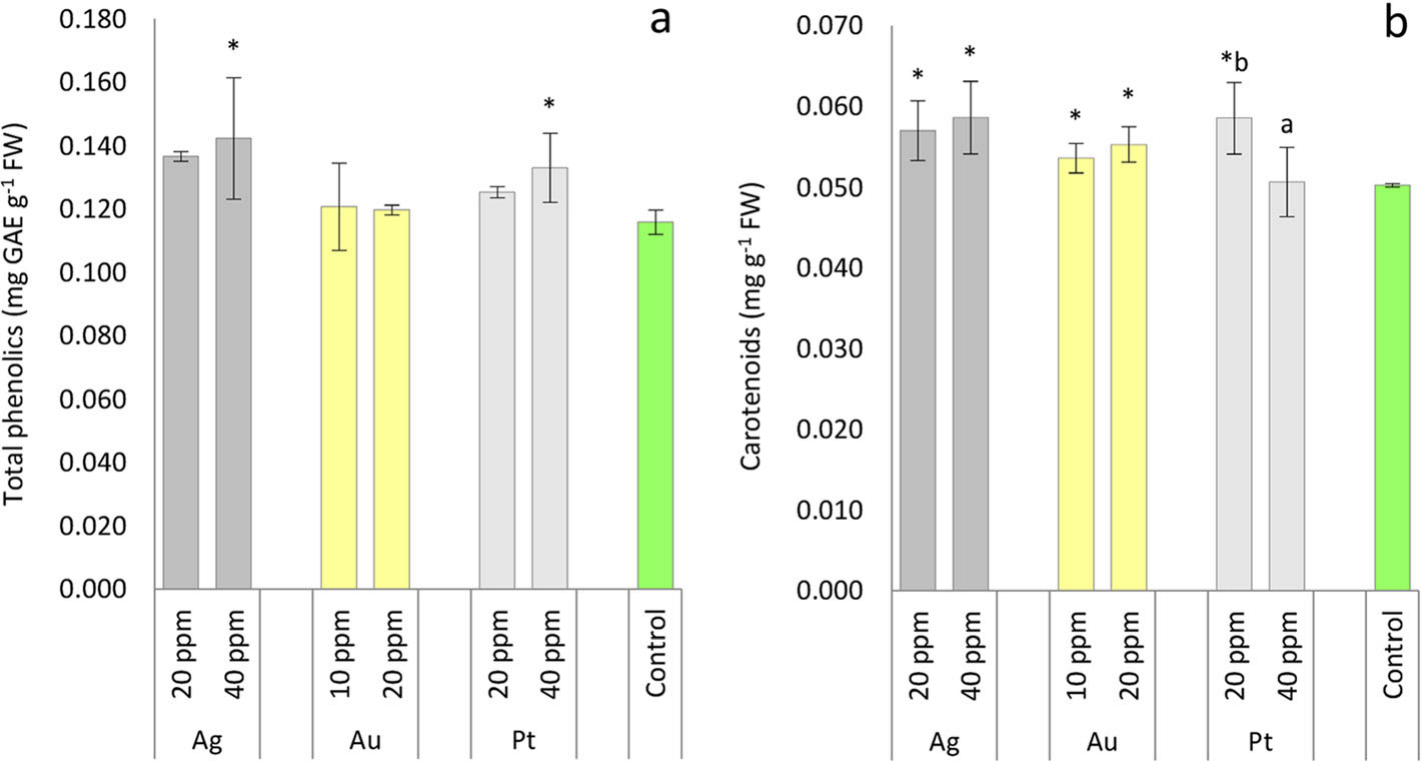
Content of total phenolics (**a**) and carotenoids (**b**) in oakleaf lettuce seedlings affected by Ag, Au and Pt nanoparticles applied to the leaves in different concentrations as aqueous colloidal solutions. Control plants were sprayed with deionized water. *Denotes significant differences (*p* ≤ 0.05) between particular nanometal and unexposed control, means for concentration of given nanometal with no letters are not significantly different at *p* ≤ 0.05, comparisons were performed by Fisher’s LSD test. Bars represent standard deviations (± SD)

The results of the total phenolics study revealed that Ag-NPs and Pt-NPs at 40 ppm concentration increased phenolics content by 17 and 15%, respectively, compared to the control (Fig. 3a). No response of oakleaf lettuce seedlings to Au-NPs, 20 ppm Ag-NPs and 20 ppm Pt-NPs was observed in respect of phenolics concentration. Changes in carotenoids content at 40 ppm Pt-NPs treatments were negligible (Fig. 3b). However, Ag-NPs increased carotenoids contents in oakleaf lettuce when 20 ppm and 40 ppm solutions were applied (by 13 and 17%, respectively, compared to control), and 20 ppm Pt-NPs caused an increase in carotenoids concentrations (by 16.5%), moreover, this value was significantly higher than that of 40 ppm Pt-NPs. We also observed an increase in carotenoids level after spraying plants with Au-NPs (by 7 and 10%, respectively for 10 and 20 ppm) compared to the control group. Studies using *Bacopa monnieri* proved that Ag-NPs (10 ppm, hydroponic culture) increased the total phenolics content in the plant organs [35]. Foliar spraying of *Echium amoenum* with 20 and 50 ppm solutions of Ag-NPs significantly increased total phenolics content compared to control seedlings [39]. Also, Najafi et al. [40] observed that Ag-NPs (50 ppm) caused an increase in total phenolics in *Triticum aestivum* seedlings. Judging by the data reported in the literature, there is generally a positive correlation between phenolics content and plant exposure to Ag-NPs, in our case such a relationship was revealed at higher Ag-NPs concentrations. In the present experiment we also observed an increase in phenolics content due to 40 ppm Pt-NPs application; Astafurova et al. [29] found that treatment of wheat seedlings with Pt-NPs led to an increase in flavonoids content – depending on the type of soil in which the plants grew, this increase even reached 40% compared to the control. Mirzajani et al. [25] observed a significant increase in carotenoids content in rice shoots when plants were treated with 60 mg L^− 1^ Ag-NPs, which is consistent with our data. However, Larue et al. [27] reported no response of lettuce seedlings in respect of carotenoids content when different concentrations of Ag-NPs were applied to the plants and, moreover, Vishwakarma et al. [41] reported a decrease in carotenoids content in mustard treated with 1 mM and 3 mM Ag-NPs. It is worth emphasizing that the level of carotenoids in plants increased due to the use of lower Pt-NPs concentration and both concentrations of Au-NPs and Ag-NPs. It seems that increasing of carotenoids content after M-NPs treatment is to protect plants against oxidative stress, together with phenolic compounds, but in the case of phenolics higher concentrations of Ag and Pt nanoparticles were necessary to induce significant response.

**Fig. 4:**
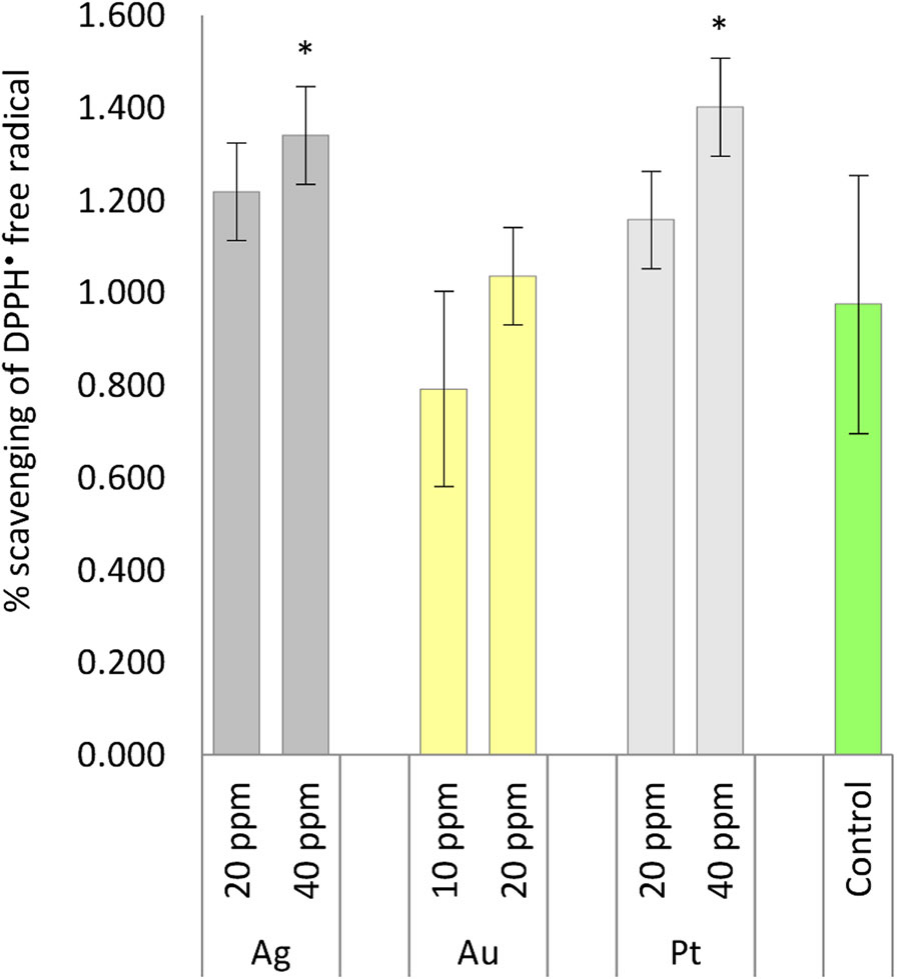
Total antioxidant capacity of oakleaf lettuce seedlings affected by Ag, Au, and Pt nanoparticles applied to the leaves in different concentrations as aqueous colloidal solutions. Control plants were sprayed with deionized water. *Denotes significant differences (*p* ≤ 0.05) between particular nanometal and unexposed control, means for concentration of given nanometal with no letters are not significantly different at *p* ≤ 0.05, comparisons were performed by Fisher’s LSD test. Bars represent standard deviations (± SD)

Plants treated with 40 ppm of Ag-NPs and Pt-NPs showed significantly higher total antioxidant capacity when compared to control (Fig. 4). Scavenging of DPPH radicals by extracts of NPs-treated plants was higher by 37.5 and 44%, respectively, than the control. No effects of lower concentrations of Ag-NPs, Pt-NPs, or both concentrations of Au-NPs on this trait was observed. As described for *Corchorus olitorius*, antioxidant activity increased in a dose-dependent manner in accordance with increasing Ag-NP concentrations in soil [42]. Abbasi and Jamei [39] noted that DPPH free radical scavenging activity was significantly higher in *Echium amoenum* seedlings after foliar spraying with 50 ppm Ag-NPs, but not with 20 ppm Ag-NPs, which agrees with our findings. Kumar et al. [28] showed, on the basis of DPPH assay results, that total free radical scavenging activity was improved in *A. thaliana* seedlings grown in a medium with Au-NPs compared to the control, but we did not observe such a relationship in the present experiment. To the best of our knowledge, there is no information available regarding the measurement of antioxidant activity in plant samples treated with Pt-NPs, thus an increase in DPPH scavenging activity in oakleaf lettuce treated with 40 ppm Pt-NPs is an interesting result of our experiment. The positive correlation between total phenols content and activity in scavenging the DPPH radicals, was observed in our experiment and reflected by the data presented in Figs. 3 and 4.

According to the results, there was a significant effect of Ag-NPs on chlorophyll *a* content in oakleaf lettuce seedlings, when Ag-NPs was applied at 20 and 40 ppm concentrations, which led to an increase in the quantity of this pigment (Table 2). All tested concentrations of Au-NPs and Pt-NPs applied to the plant leaves did not markedly affect chlorophyll *a* content in comparison to control. No nanoparticle treatments changed the content of chlorophyll *b* in the seedlings. Salama [23] increased concentrations of Ag-NPs from 20 to 60 ppm, which led to an increase in chlorophyll content in common bean (by 49% for chlorophyll *a* and 33% for chlorophyll *b*) and corn seedlings (by 46 and 26%, respectively) above the control, while Ag-NPs concentrations above 60 ppm caused degradation of chlorophyll pigments. Mirzajani et al. [25] found the highest concentration of chlorophyll *a* when 60 mg L^− 1^ Ag-NPs was applied to rice plants; however, degradation of chlorophyll *b* was observed. Sharma et al. [34] noted higher chlorophyll content in leaves of *B. juncea* seedlings treated with Ag-NPs, as compared to the control seedlings. These authors reported improved quantum efficiency of the Ag-NPs-treated seedlings which indicated that higher number of reaction centres were in an ‘open state’ to carry out light reaction. It decreases probability of generation of reactive radicals, damages to the chloroplasts and destruction of chlorophylls. Our results also showed that among non-enzymatic antioxidants mainly carotenoids responded to Ag-NPs treatment, and the role of these pigments in protecting chlorophylls from destruction under stress conditions is known. Nair and Chung [36] showed that total chlorophylls content of *A. thaliana* seedlings did not change after exposure to 0.2 mg L^− 1^ of Ag-NPs as compared to the control seedlings but the chlorophyll content decreased after exposure to 0.5 and 1 mg L^− 1^ of Ag-NPs. When three concentrations of Ag-NPs were tested on lettuce seedlings by Larue et al. [27] no alteration in chlorophyll pigment content occurred due to the treatments. In our experiment we did not observe any significant effects of Au-NPs on chlorophyll concentration, but it has been reported that treatment with Au-NPs (10–100 ppm) could produce higher chlorophyll content in *B. juncea* seedlings, especially in seedlings treated with 10 ppm Au-NPs [13]. No response of wheat seedlings in respect of chlorophyll level after exposure to Pt-NPs was observed by Astafurova et al. [29].

**Table 2:** Chlorophyll *a* and chlorophyll *b* content of oakleaf lettuce seedlings depending on engineered nanoparticles (nano-metals; M-NPs) applied to the leaves in different concentrations as aqueous colloidal solutions. Control plants were sprayed with deionized water

M-NPs and concentration		Chlorophyll *a* (mg g^− 1^ FW)	Chlorophyll *b* (mg g^− 1^ FW)
Ag	20 ppm	0.274 ± 0.007 *	0.105 ± 0.008
Ag	40 ppm	0.283 ± 0.020 *	0.106 ± 0.009
Au	10 ppm	0.256 ± 0.010	0.102 ± 0.007
Au	20 ppm	0.269 ± 0.046	0.107 ± 0.014
Pt	20 ppm	0.267 ± 0.015	0.100 ± 0.002
Pt	40 ppm	0.247 ± 0.017	0.107 ± 0.020
Control		0.243 ± 0.007	0.094 ± 0.007

*Denotes significant differences (*p* ≤ 0.05) between particular nanometal and unexposed control, means for concentration of given nanometal with no letters are not significantly different at *p* ≤ 0.05, comparisons were performed by Fisher’s LSD test. Each value represents the mean ± SD

The metal content of oakleaf lettuce seedlings increased with increasing concentrations of M-NPs applied to the plants (Table 3). Very small amounts of these metals were detected in control seedlings. Plants treated with 20 ppm Ag-NPs and 40 ppm Ag-NPs had 114 and 138 times more Ag compared to the control. In the case of Pt, these values were 75 and 95 times more, respectively. Au-NPs-treated plants had 19.5 and 23 times more Au (for 10 and 20 ppm, respectively) than control seedlings. Twice the concentration of M-NPs solution applied to plants did not translate into a doubling in the amount of given metals in tissues. Positive relationships between metal contents in the plants and an increase in NPs concentration supplied to plants is in agreement with literature data. Applying Ag-NPs with increasing solution concentrations (2, 10, 20 mg L^− 1^) led to a proportional increase in the content of this element in potato plantlets [33]. Torrent et al. [43] described total Ag accumulated in lettuce root tissues increasing in dose-dependent manner for Ag-NPs, but in the case of shoots such an increase was observed only up to the concentration 7 mg L^− 1^ in the growing medium. Feichtmeier et al. [14] observed that the Au content in barley roots rose with increasing Au concentration in the nutrient medium up to 8 μg Au mL^− 1^, but at the highest exposure concentration of 10 μg Au mL^− 1^, slightly lower values occurred. Asztemborska et al. [15] showed that the Pt content of *L. sativum* and *S. alba* shoots was clearly dependent on the Pt-NPs in the growth medium and for the highest Pt-NPs concentration applied (100 mg L^− 1^), it reached the highest level in the plants.

**Table 3:** Content of Ag, Au, Pt (ppb in extracts) in oakleaf lettuce seedlings depending on engineered nanoparticles (nano-metals; M-NPs) applied on the leaves in different concentrations as aqueous colloidal solutions. Control plants were sprayed with deionized water

M-NPs and concentration		Elements content
		Ag
Control		0.007 ± 0.003
Ag	20 ppm	0.799 ± 0.182 *
Ag	40 ppm	0.966 ± 0.124 *
		Au
Control		0.022 ± 0.005
Au	10 ppm	0.428 ± 0.127 *
Au	20 ppm	0.508 ± 0.044 *
		Pt
Control		0.001 ± 0.000
Pt	20 ppm	0.070 ± 0.016 *
Pt	40 ppm	0.095 ± 0.025 *

*Denotes significant differences (*p* ≤ 0.05) between particular nanometal and unexposed control, means for concentration of given nanometal with no letters are not significantly different are significantly different at *p* ≤ 0.05, comparisons were performed by Fisher’s LSD test. Each value represents the mean ± SD

**Fig. 5:**
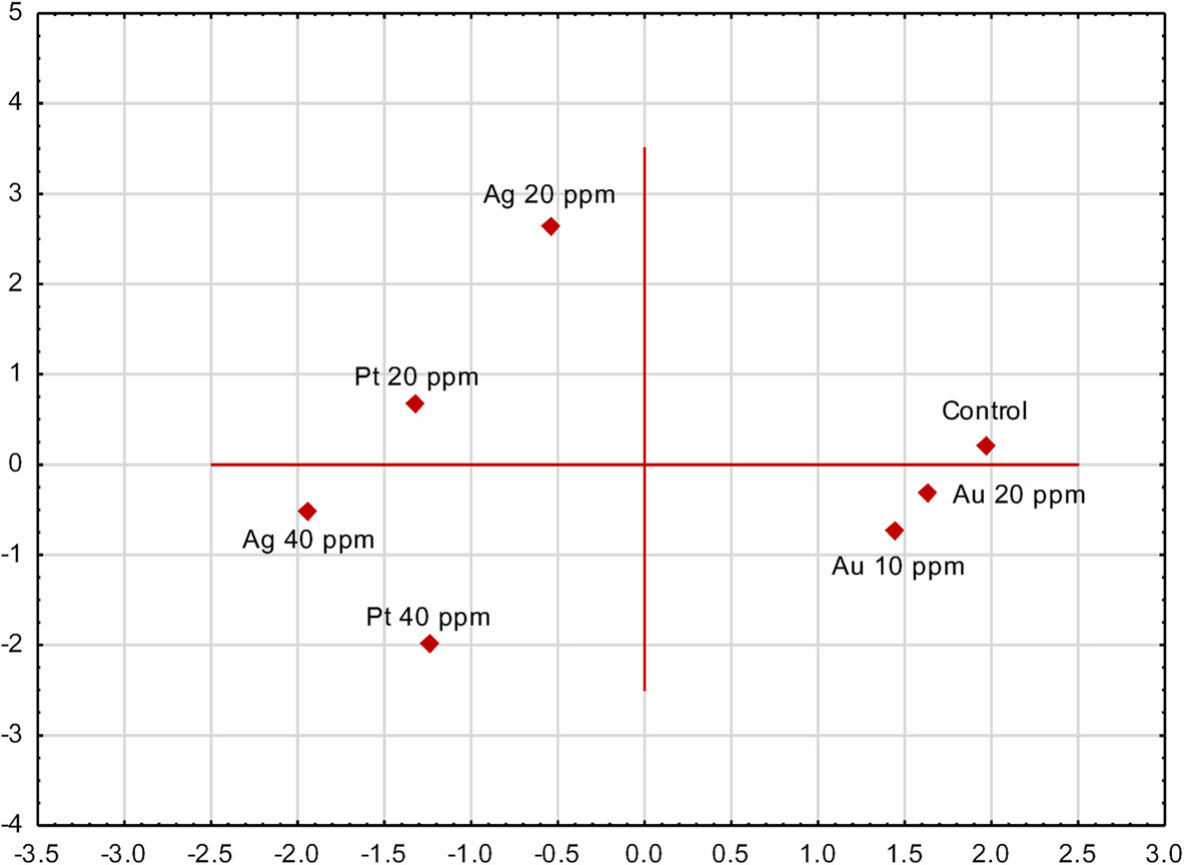
Ordination diagram obtained by principal component analysis (PCA) showing similarities among experimental treatments according to enzymatic antioxidant activity and non-enzymatic antioxidant compounds content in oakleaf lettuce seedlings

PCA was used to investigate the effects of foliar exposure of oakleaf lettuce seedlings to M-NPs applied at different concentrations on the content of non-enzymatic antioxidants and the activity of antioxidant enzymes (Fig. 5). The data revealed that PC1 and PC2 accounted for 77.33% of the total variance within the data set, contributing 38.02 and 29.31%, respectively. On the basis of the factor loading values, it may be concluded that the first component mainly represents the control, which was placed alone in the upper right plot (both loadings positive) and Au-NPs (PC1 loadings positive, but PC2 loadings negative). The second component is connected mostly with the 20 ppm Ag-NPs treatment and, to a lesser degree, with the 20 ppm Pt-NPs treatment (for both treatments there were negative factor loadings associated with the first component, and positive loadings with the second component). Higher concentrations of Ag-NPs and Pt-NPs were placed together in the left lower plot, with both loadings negative. The short distance between the control and Au-NPs confirmed the rather small impact of Au-NPs on the antioxidant status of plants, essentially this effect of Au-NPs occurred only in the case of total POX activity. The differences between 20 ppm and 40 ppm of Ag and Pt that can be seen in Fig. 5 resulted from the often higher content of antioxidants when higher concentration of nanoparticles was used, although these differences were not always statistically confirmed through ANOVA analysis.

## Conclusions

Results in this study have shown that foliar exposure of oakleaf lettuce to M-NPs (Ag, Au and Pt) of different concentrations modulated biochemical processes in the seedlings in different way. Among the tested nanoparticles, Au-NPs increased total POX activity, glutathione and carotenoids concentration, but it did not affect the content of other non-enzymatic antioxidants and APX activity. Glutathione content increased due to Pt-NPs application, but L-ascorbic acid content was unaffected by M-NPs in comparison to control plants. Ag-NPs increased carotenoids content together with 20 ppm Pt-NPs, total phenolic content increased when Ag-NPs and Pt-NPs were applied, but only at 40 ppm concentration. The higher concentration (40 ppm) of Ag-NPs and Pt-NPs caused the greatest increase in DPPH radical scavenging activity compared to control. Judging by the lack of significant changes in fresh and dry weight caused by M-NPs (40 ppm Pt-NPs caused even an increase in FW) and the lack of visible negative changes in plants, it should be stated that the concentrations of M-NPs used were not toxic to the plants. However, applied M-NPs influenced plant metabolism, specific to the nanoparticle treatment. It should be emphasized that positive or negative impacts of nanoparticles on plants are known, but the literature data are contradictory in this regard. A coordinated research program incorporating standardized experimental procedures, including concentrations, growing medium for the plants, NPs application path (root or foliar exposure), ontogenetic stage of the plant, etc. seems to be required.

## Methods

### Nanoparticles and their characteristics

Nanoparticles of silver (Ag-NPs), gold (Au-NPs), and platinum (Pt-NPs) were used in the experiment. Nanometals were purchased from PlasmaChem GmbH (Berlin, Germany) as aqueous colloidal solution, obtained in the form of ca. 0.10 mg cm^− 3^ (Ag) and 0.05 mg cm^− 3^ (Au) colloidal solution in water with citrate as stabilizer. Pt was supplied as a dry powder, but forms an aqueous colloidal solution (0.10 mg Pt cm^− 3^) in water with polyvinyl pyrrolidone (PVP) stabilizer. Average Ag particle size was ca. 10 nm, Au was ca. 20 nm, and Pt was ca. 3 nm. A series of treatment solution was prepared with deionized water to obtain concentrations of 20 and 40 ppm of Ag and Pt, and 10 and 20 ppm of Au. All beakers were placed together in a room at a temperature of about 22 °C in the daytime (16 h) and 18 °C at night (8 h).

### Plant material and nanoparticle application

Seedlings of oakleaf lettuce (*Lactuca sativa* L. var. *foliosa* Bremer) cv. Kiribati (seeds supplied by Rijk Zwaan Polska Sp. z o.o., Warsaw, Poland) were purchased from Krasoń – A Group of Vegetable Seedling Producers (Piaski, Poland). Seedlings were grown at 18/15 °C (day/night) in cubic peat pots of 64 cm^3^ volume placed in plastic boxes (150 pots per plastic box). Goëmar Goteo (Arysta LifeScience Polska Sp z o. o., Warsaw, Poland) was applied to the seedlings as a biofertilizer at the two-leaf stage via a single foliar spray at a concentration of 0.3%. Two-week-old seedlings (4–5 leaves) were transferred to University of Agriculture in Kraków greenhouse and placed on a table, then the plants were irrigated by flooding the table (up to ¾ height of the pots), as required. Nanoparticles of Ag, Au and Pt were applied only once, evenly to the leaves two days later, at the concentrations mentioned above, a 50 cm^3^ suspension per box was applied (ca. 0.33 cm^3^ per plant). Control plants were sprayed with deionized water at the same time. No additional fertilization was used during trial. After 7 days all plants from the experimental treatments were harvested (one replicate consisted of 150 plants from one plastic box, in total three replicates were established for each treatment). All leaves were carefully washed with tap water and then rinsed with deionized water. Then all leaves from a treatment were mixed, laboratory samples were taken from these leaves and inserted into ultra-deep freezer to a temperature of − 40 °C for further analyses. For shoot fresh and dry weight determination, exactly 15 plants per replicate were sampled.

### Leaf fresh and dry weight

Leaf rosettes of each individual plant were weighed with a Sartorius A120S balance (Sartorius AG, Göttingen, Germany) to determine fresh weight (FW) per plant. Dry weight (DW) was measured by drying samples at 65 °C in an oven until constant weight was obtained. Total dry weight content of plant aerial part (shoot) was presented as average values expressed in grams.

### Chlorophyll and carotenoids quantification

Chlorophyll *a*, chlorophyll *b*, and carotenoids were measured, according to the procedure described by Lichtenthaler and Wellburn [44], by extracting 0.1 g of fresh leaf sample in 25 cm^3^ of 80% (*v*/*v*) acetone using 3 mg of magnesium carbonate (MgCO_3_) as a pigment stabilizer. After 0.5 h incubation in the dark, the suspension obtained was filtered through a filter paper (POCH SA, No. 978774513, Gliwice, Poland). Absorption of the extracts was measured using a spectrophotometer (UV-VIS Helios Beta, Thermo Fisher Scientific Inc., Waltham, USA) at 646, 663, and 470 nm to quantify the chlorophyll *a*, chlorophyll *b*, and total carotenoid content, respectively, based on the equations reported by Lichtenthaler and Wellburn [44].

### Antioxidant enzyme extraction and assay

Peroxidase (POX, EC 1.11.1.7) activity was expressed as an increase of absorbance of *p*-phenylenediamine oxidized to phenazine by enzymes from plant tissue [45]. Two grams of plant sample were ground in an ice-bath (4 °C) in 10 cm^3^ of solution containing 0.05 M potassium phosphate buffer (pH 7.0). After 2 min, an additional 5 cm^3^ buffer was added. The mixture was centrifuged at 3492 g for 15 min at 4 °C, and the supernatant was used for enzyme assay. The reaction mixture consisted of supernatant, 0.05 M potassium phosphate buffer, *p*-phenylenediamine, and H_2_O_2_ solution. Absorbance was measured at 485 nm at 60-s intervals for 2 min on a UV-VIS Helios Beta spectrophotometer. One unit (U) of enzyme activity was expressed as the increase of absorbance by 0.1 for 1 min.

The procedure of ascorbate peroxidase (APX, EC 1.11.1.11) determination started with preparing the mixture of 4 g of leaf samples that were homogenized in 4 °C with 10 cm^3^ 50 mM potassium phosphate buffer (pH 7.0) with 1 mM ethylenediaminetetraacetic acid (EDTA), 1% soluble polyvinylpyrrolidone (PVP), and 1 mM phenylmethylsulfonyl fluoride (PMSF). The mixture was centrifuged at 13968 g for 15 min at 4 °C, and the supernatant was used for enzyme assay. APX activity was measured as a decrease in absorbance at 290 nm for 5 min [46]. The assay mixture consisted of 0.5 mM ascorbate, 0.1 mM H_2_O_2_, 50 mM potassium phosphate buffer (pH 7.0), and 0.15 cm^3^ of enzyme extract. Activity of the enzyme was quantified using the molar extinction coefficient for ascorbate (ε = 2.8 mM^− 1^ cm^− 1^) and expressed as μg AsA min^− 1^ g^− 1^ FW.

### Determination of glutathione and L-ascorbic acid

The reduced form of glutathione (GSH) was extracted and determined according to the method described by Guri [47] with modifications. Fresh leaves (2.5 g) were homogenized in an ice-bath (4 °C) with 6 cm^3^ 0.5 mM EDTA and 3% trichloroacetic acid (TCA). After centrifugation at 6208 g for 10 min at 4 °C, K-phosphate buffer was added to bring the pH to 7.0 and Ellman’s reagent (5,5-dithiobis-2-nitrobenzoic acid, DTNB) was added to the supernatant. The reaction was monitored as the rate of change in absorbance at 412 nm on UV-VIS Helios Beta spectrophotometer against a blind sample (mixture of 2.0 cm^3^ of plant homogenate and 1.0 cm^3^ 0.2 M K-phosphate buffer). Calculations were made on the basis of a standard curve, and content was expressed as μg g^− 1^ FW.

L-ascorbic acid content was measured according to Krełowska-Kułas [48] with Tillman’s titration method. Fresh leaves (12.5 g) were homogenized in ice-cold 50 cm^3^ acetic acid and after 30 min the mixture was titrated with Tillman’s reagent (2,6-dichlorophenol-indophenol) until the colour turned pink. The titration volume was used for calculation of the L-ascorbic acid concentration, which was expressed in mg 100 g^− 1^ FW.

### Total phenolics content

Total phenolics were determined according to the Folin-Ciocalteu colorimetric method described by Djeridane et al. [49]. Two grams of fresh plant material was mixed with 10 cm^3^ of 80% methanol and then centrifuged at 3492 g for 10 min. The sample (0.1 cm^3^) was dissolved in 2 cm^3^ of sodium carbonate (Na_2_CO_3_), 1.5 ml distilled water and 0.1 cm^3^ Folin-Ciocalteu’s reagent and deionized water (1:1 *v*/*v*). The final mixture was shaken and incubated for 45 min in the dark at 22 °C. The absorbance of the mixture was measured at 750 nm using the UV-VIS Helios Beta spectrophotometer. A standard curve was plotted using gallic acid as a standard. Results were expressed as milligrams of gallic acid equivalents (GAE) per gram FW (mg of GAE g^− 1^ FW).

### DPPH^•^ radical scavenging activity

The DPPH radical scavenging ability of samples was monitored according to the method described by Molyneux [50]. The absorbance was recorded at 517 nm on a UV-VIS Helios Beta spectrophotometer. Two and a half grams of ground plant material in 80% methanol were centrifuged (3492 g, 10 min, 4 °C). The assay mixture consists of 0.1 mL of supernatant and 4.9 mL of 0.1 mM DPPH^•^ dissolved with 80% methanol. The mixture was shaken in a vortex mixer and incubated at 20 °C in the dark for 15 min. Inhibition of free radicals by DPPH was calculated using the following equation:

$$ \mathrm{AA}\ \left[\%\right]=\left[\left({\mathrm{A}}_0-{\mathrm{A}}_1\right)/{\mathrm{A}}_0\right]\times 100 $$

where AA is the antioxidant activity, A_0_ is the absorbance of the control solution, and A_1_ is the absorbance of the test solution.

### Ag, Au, and Pt content

The procedure for determining the elements is described by Pasławski and Migaszewski [51] and Kalisz et al. [52]. Briefly, randomly-selected lettuce leaves were shredded and dried at 70 °C in a dryer. The dried samples were ground using a Pulverisette 14 ball mill (Fritsch GmbH, Idar-Oberstein, Germany; 0.5-mm sieve). After that, 3 g samples were placed in TFM vessels with a volume of 100 cm^3^ and mineralized in 10 cm^3^ 65% super pure HNO_3_ (Merck no. 100443.2500) in a Mars 5 Xpress (CEM Corporation, Matthews, NC, USA) microwave digestion system. After cooling, the samples were transferred to 25 cm^3^ flasks with redistilled water. The total contents of the elements Ag, Au, and Pt were analysed by ICP-MS/MS triple quadruple spectrometer iCAP TQ ICP-MS (Thermo Fisher Scientific Inc., Bremen, Germany). Their determination was conducted using the following measurement mode for individual isotopes of elements: S-SQ-KED for ^197^Au, ^109^Ag, and ^195^Pt.

### Data analysis

The results were expressed as means (*n* = 3) ± SD (standard deviation). Statistical analysis was performed with the Statistica 13.3 package (TIBCO Software Inc., Palo Alto, CA, USA). Differences between particular nanometal and untreated control were analysed using one-way ANOVA and Fisher’s LSD post-hoc test. A *p*-value of less or equal than 0.05 was considered to be statistically significant. Principal component analysis (PCA) was carried out for the NPs and antioxidants studied. Data for the activity of antioxidant enzymes and non-enzymatic antioxidant compound content were standardized before the analytical procedure. PCA analysis was performed using Statistica 13.3 and the first two components (PC1 and PC2) were used to make biplots.

## Data Availability

Most data supporting the results are included in the article. The datasets used and/or analysed during the current study are available from the corresponding author on reasonable request.

## Abbreviations

Ag-NPs: Silver nanoparticles
APX: Ascorbate peroxidase
AsA: Ascorbate
AsA-GSH: Ascorbate-glutathione cycle
Au-NPs: Gold nanoparticles
DPPH: Radical scavenging activity
DW: Dry weight
FW: Fresh weight
EDTA: Ethylenediaminetetraacetic acid
GAE: Gallic acid equivalents
GPX: Guaiacol peroxidase
GSH: Reduced glutathione
M-NPs: Metal nanoparticles
NPs: Nanoparticles
PCA: Principal component analysis
PMSF: Phenylmethylsulfonyl fluoride
POX: Peroxidase
Pt-NPs: Platinum nanoparticles
PVP: Polyvinylpyrrolidone
ROS: Reactive oxygen species
TCA: Trichloroacetic acid
